# Monkeypox genome mutation analysis using a timeseries model based on long short-term memory

**DOI:** 10.1371/journal.pone.0290045

**Published:** 2023-08-23

**Authors:** Refat Khan Pathan, Mohammad Amaz Uddin, Ananda Mohan Paul, Md. Imtiaz Uddin, Zuhal Y. Hamd, Hanan Aljuaid, Mayeen Uddin Khandaker

**Affiliations:** 1 Department of Computing and Information Systems, School of Engineering and Technology, Sunway University, Selangor, Malaysia; 2 Department of Computer Science and Engineering, Chittagong University of Engineering & Technology, Chittagong, Bangladesh; 3 Department of Computer Science and Engineering, BGC Trust University Bangladesh, Chittagong, Bangladesh; 4 Department of Pharmacy, State University of Bangladesh, Dhaka, Bangladesh; 5 Department of Radiological Sciences, College of Health and Rehabilitation Sciences, Princess Nourah bint Abdulrahman University, Riyadh, Saudi Arabia; 6 Computer Sciences Department, College of Computer and Information Sciences, Princess Nourah bint Abdulrahman University (PNU), Riyadh, Saudi Arabia; 7 Centre for Applied Physics and Radiation Technologies, School of Engineering and Technology, Sunway University, Selangor, Malaysia; 8 Department of General Educational Development, Faculty of Science and Information Technology, Daffodil International University, Dhaka, Bangladesh; Lagos State University, NIGERIA

## Abstract

Monkeypox is a double-stranded DNA virus with an envelope and is a member of the Poxviridae family’s Orthopoxvirus genus. This virus can transmit from human to human through direct contact with respiratory secretions, infected animals and humans, or contaminated objects and causing mutations in the human body. In May 2022, several monkeypox affected cases were found in many countries. Because of its transmitting characteristics, on July 23, 2022, a nationwide public health emergency was proclaimed by WHO due to the monkeypox virus. This study analyzed the gene mutation rate that is collected from the most recent NCBI monkeypox dataset. The collected data is prepared to independently identify the nucleotide and codon mutation. Additionally, depending on the size and availability of the gene dataset, the computed mutation rate is split into three categories: Canada, Germany, and the rest of the world. In this study, the genome mutation rate of the monkeypox virus is predicted using a deep learning-based Long Short-Term Memory (LSTM) model and compared with Gated Recurrent Unit (GRU) model. The LSTM model shows “Root Mean Square Error” (RMSE) values of 0.09 and 0.08 for testing and training, respectively. Using this time series analysis method, the prospective mutation rate of the 50^th^ patient has been predicted. Note that this is a new report on the monkeypox gene mutation. It is found that the nucleotide mutation rates are decreasing, and the balance between bi-directional rates are maintained.

## Introduction

The monkeypox virus (MPV) is a smallpox-related orthopox DNA virus from the Poxviridae family [1–3]. It is currently causing worry on a global scale. In Central and West Africa, two distinct genetic subtypes are recognized to be disease-causing. Compared to the Central African subtype, the disease is less acute in the West African subtype [4]. The natural reservoirs are probably different African rodents and primates [5]. The monkeypox virus (MPV) can be transmitted to anyone by close, direct, and frequent skin-to-skin contact in several ways. Human-to-human transmission may result from close physical contact with an infected person or animal through respiratory droplets, bodily fluids, lesions, and contaminated objects like bedding [5, 6]. Direct contact with infected animals through scratches and bites zoonosis spread. Rather than monkeys, rodents like mice, rats, and squirrels carry the disease, which is then passed on to humans [4]. The invasion period of monkeypox lasts between 0–5 days, the affected people usually experience viral symptoms like fever, tiredness, headaches, general achiness followed by developing a rash, while some other affected people experience them after the rash appears. Until the rash is entirely gone and a new layer of skin has formed on the sick person, monkeypox is communicable from the time when the symptoms start to manifest. It usually lasts typically 2 to 4 weeks [4–6]. Recently, the patient mortality rate ranged from 3–6%. In contrast to COVID-19, this virus doesn’t travel from person to person effectively. It is also much simpler to isolate affected people and stop the transmission. Through the placenta, a pregnant individual can transmit the virus to their unborn child, which is too dangerous for the child. Not only this, air travelers are crucial in the spread of sickness [7, 8].

Monkeypox was initially detected in colonies of monkeys held for research in 1958 following two outbreaks of a condition resembling pox [9, 10]. The disease monkeypox virus was first discovered in a human being in 1970. Infection outbreaks have been observed sporadically in Africa, usually due to interaction with wildlife reservoirs (mainly rodents) [11]. Nearly all occurrences of monkeypox infection in people outside of Africa before the 2022 outbreak were connected to either imported animals or international travel to countries [9]. In 2003, the monkeypox outbreak was reported for the first time outside of Africa, where pet prairie dogs were infected because those dogs were housed with dormice imported from Ghana and Gambian pouched rats [6, 11]. Along with this, the United States confirmed more than 70 cases of monkeypox in the same year. Travelers reported numerous instances of monkeypox from Nigeria to other nations, including the United Kingdom (UK) in 2018–19, 2021–22, Israel in 2018, Singapore in 2019, and the United States (US) in 2021 [6]. In Nigeria, 76 cases were reported in 2018, of which 37 are confirmed, one is likely, and two have resulted in death [12]. More monkeypox cases were reported in many non-endemic nations in May 2022. Interestingly, the monkeypox virus strain causing the current epidemic of the disease in nonendemic states probably branched from the monkeypox virus that caused an outbreak in Nigeria in 2018–19 and has far more mutations than would be anticipated, some of which increase transmission [13]. Since early May 2022, more than 50 countries across five regions have reported over 3000 instances of the monkeypox virus infection [1]. The current monkeypox outbreak was consequently classified as a “Public Health Emergency of International Concern” by the "World Health Organization" (WHO) on 23rd July 2022 [14]. According to data from around the world, most cases of the current monkeypox outbreak are among gays and bisexuals [15].

Mutation analysis has been a hot topic since the occurrence of COVID-19 in 2019, and now whenever the outbreak of any potential pandemic capable disease happens, the first question that come around to our mind is if it can change or evolve with time. This research also gets the motivation from the adaptation characteristics of monkeypox. And we wanted to analyze not only the past mutation rates but also the future rates with the help of machine learning. In this paper, we worked with the gene mutation which is almost new in regard to the monkeypox gene mutation. Furthermore, we have processed big data which is not done for the recent pandemic such as COVID-19. This paper is mainly focused on the general readers so that people with no background of mutation rate studies can understand how machine learning is used in mutation analysis related tasks. The major contribution of this study is as follows:

1. We analyzed the genome sequence based on codon and nucleotide separately.
2. Analyzed the mutation rate with own designed algorithm and created a timeseries dataset from that.
3. Trained LSTM and GRU model for future rate predictions.

This paper will analyze monkeypox’s genetic data to identify the gene mutation rate. Here, "genetic data" refers to DNA and an organism’s genome, which is the terminology usually used in bioinformatics. The rest of the paper is sectioned as literature review to discuss current word in this filed, working procedure would explain the whole workflow and dataset processing, gene mutation section would discuss the different kind of mutation rates, next section would discuss the model analysis and predictions and finally we discussed the result and concluded our work.

## Literature review

Monkeypox was declared to have developed in 2022, posing a new global health disaster, according to the WHO, after the global effects of COVID-19 in 2019 [16]. Despite it having recently occurred, Monkeypox is not the subject at hand because there has been so little research on gene mutation. The phrase "gene mutation" describes a change in one or more genes that has the potential to lead to various diseases or disorders. Time series work or forecasting any disease or its gene mutation rate is one of the great works in the field of research. In the recent past, much work has been done on COVID-19 forecasting. To predict the COVID-19 virus’s future mutation rate, a LSTM model was used in ref. [16]. The nucleotide mutation rate of the 400^th^ patient was accurately predicted by this model, which had a RMSE of 0.06 during testing and 0.04 during training. Five deep learning algorithms, including the recurrent neural network (RNN), gated recurrent units (GRUs), variational autoencoder (VAE), LSTM, and bidirectional LSTM (BiLSTM), were applied for the global forecasting of COVID-19 cases [17]. The results show that the VAE outperformed all other models in terms of forecasting performance. Besides, an extension of the RNN as an LSTM cell and its variants, such as Bi-directional LSTM, Convolutional LSTM, and Stacked LSTM adopted to forecast the Covid-19 cases for one month in the future [18]. In addition to monthly instances, LSTM models are used to forecast the number of new COVID-19-positive cases for daily and weekly purposes in all states of India [19]. The suggested strategy performed well, with errors for daily predictions of about 3% and for weekly predictions of under 8%. In order to predict the risk category, a shallow LSTM-based neural network was developed, where the trend data and meteorological data were combinedly used as input for the prediction. [20]. In ref. [21], authors proposed a deep learning-based LSTM approach to predict the trends and possible stopping time of the current COVID-19 outbreak in Canada and worldwide. They also analyzed the COVID-19 virus’s transmission rates in a couple of countries such as Italy, Canada, and the USA. The results demonstrated promising predicting abilities utilizing a time series dataset.

The number of confirmed COVID-19 cases was frequently predicted by research using different time-series techniques, such as the Auto-Regressive Integrated Moving Average (ARIMA) [22]. For forecasting, statistical and artificial intelligence (AI) models were developed to forecast the daily Covid-19 cases in Egypt [23]. Prediction models have been created using ARIMA and nonlinear autoregressive artificial neural networks (NARANN), where NARANN has a 5% forecasting error. In ref. [24], the COVID-19 outbreak in India has been analyzed, and its patterns have been predicted using classic ARIMA modeling and exponential smoothing techniques. Chintalapudi et al. [25] applied the ARIMA model to forecast registered and recovered COVID-19 cases after 60 days of lockdown in Italy. According to their projection, it will be possible for recovered cases to increase by 66% and registered cases to decrease by around 35%.

Along with other methods, classical machine learning (ML) techniques also work well in time series forecasting. In the study referenced in [26], an enhanced model based on machine learning has been used to forecast the possible threat of COVID-19 in nations worldwide. Moreover, this proposed model is implemented in a cloud computing platform for more precise and immediate forecasting of the epidemic’s growth pattern. Furthermore, different supervised machine learning models such as linear regression, support vector machine (SVM), LASSO regression, and exponential smoothing (ES) are utilized in other work to predict the COVID-19 future [27]. Related to those covid works, several AI techniques using mathematical and statistical methods have been employed in the forecast of the monkeypox virus. In ref. [28], the time series analysis model ARIMA and Neural Networks were utilized to predict the cumulative cases of monkeypox virus for the 10 days. For confirmed cases, nine different forecasting models Holt–Winter’s model, Polynomial Regression, Holt’s Linear model, AR, SARIMA, MA, ARIMA, SVR, and Prophet have been utilized in [9]. The study showed that the Prophet model is the most reliable compared to the other used model where RMSE, MSE, MAE, MAPE, and the R2 score are used as the performance indicator. In ref. [29], a novel technique based on LSTM was used to predict the monkeypox infection. To improve the LSTM model’s performance and boost forecast accuracy, the BER optimization algorithm is used that optimize the parameters of the model.

Recently, due to the availability of much widely distributed datasets, researchers are now doing classifications, prognosis analysis, mutation analysis etc. The fastest way to identify monkeypox infection is via skin lesions. A PoxNet22 model has been fine-tuned to classify monkeypox from 3192 images with a precision rate of 100% [30]. Another work has been done where authors used a mobile application interface to detect monkeypox by simply capturing photos of skin lesion [31]. In the background of this mobile application, they used pre-trained EfficientNetb0 and MobileNetv2. Another broad statistical and regression analysis has been done using nine different forecasting models with global monkeypox cases dataset [10]. They find out that Spain is in a bad and Europe is in a dangerous situation. Also, they used timeseries models to predict the cases which was incremental till august 2022. So far, one mutation analysis has been found for monkeypox which worked with GenBank dataset and figured out the 2022 substitution mutation rate as 38.63 worldwide [32]. Though we have observed a significant number of studies in this field, the amount of work to analyze the monkeypox data compared with COVID-19 is pretty low.

The major gap we noticed throughout the literature is that, researchers are using daily affirmative cases of monkeypox or COVID-19 to predict future case rate. On the other hand, genetic researchers are analyzing the mutation rates for past confirmed cases. So, the future mutation calculation is missing in this scene which we addressed and analyzed in this paper.

## Working procedure

**Fig 1:**
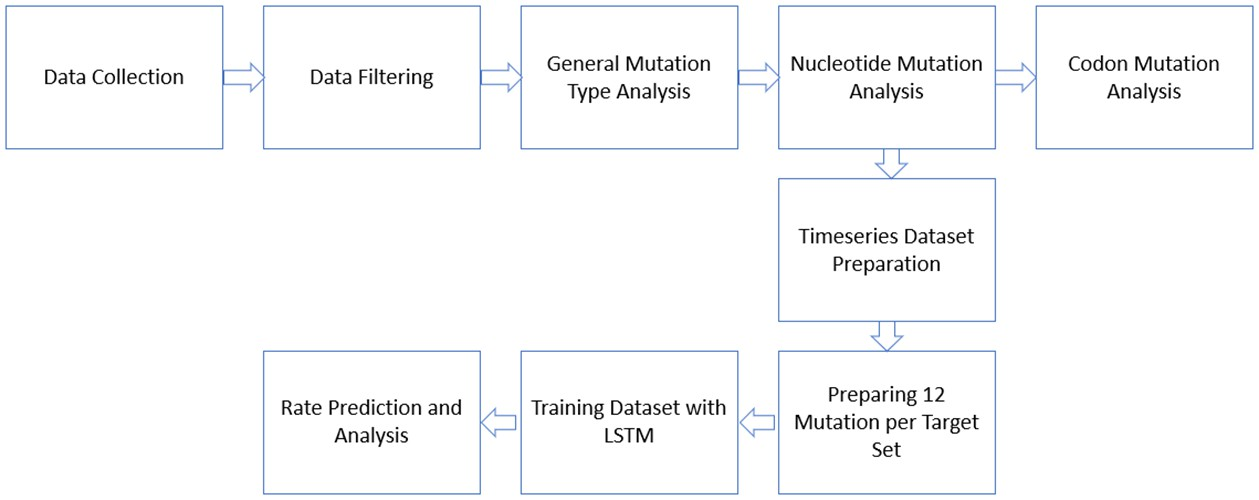
Summary illustration of whole process.

This paper is designed to represent the mutation rate analysis from a pure computer science point of view. We have collected the gene data from NCBI public database and filtered with some custom parameters (described in next section) to get the suitable genes. Next, we analyzed the missense, nonsense and silent mutations. Also, we have calculated the nucleotide mutation and codon mutation rates considering the protean transformation. From the nucleotide mutation rates, we prepared a timeseries dataset considering 12 set as 1 target value, and trained with LSTM as it is popular for its capability such as memorizing the data for a longer period in layers. We selected 12 sets, because we have 12 nucleotide transformations. The whole process is visualized in Fig 1 and each step of this process is discussed in detail in the following sections.

### Dataset preprocessing and insights

**Fig 2:**
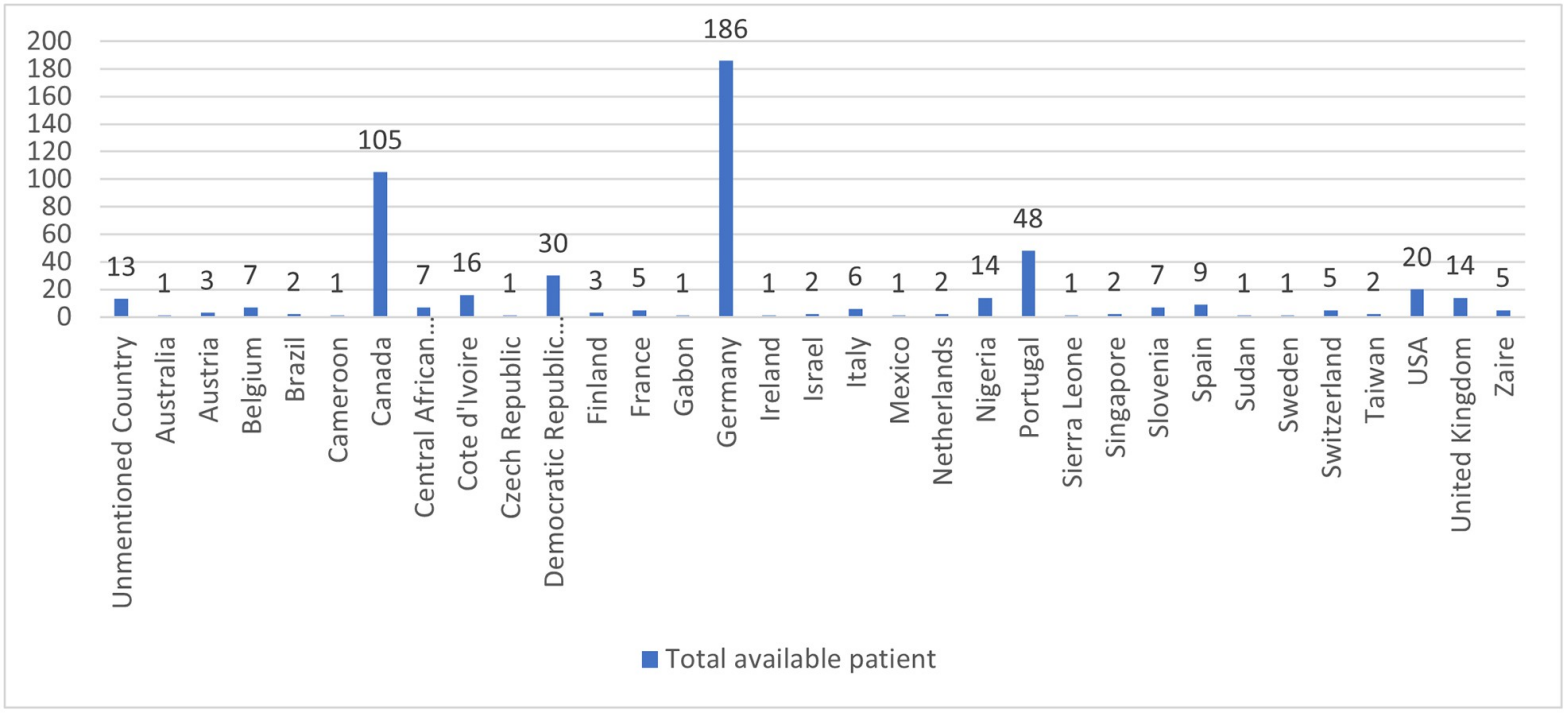
The number of patients in 33 countries.

A sufficient number of related gene datasets are publicly available in the NCBI GenBank, which contains the entire genome sequence of monkeypox. We have filtered a large number of entities using the gene sequence, sample nation, and collection date till: 24^th^ July 2022. All genes were taken from the monkeypox-affected human body. Although there are genes from almost 33 different countries, Canada and Germany have a substantial amount of patient data. To cover as many regions as possible, we have included these countries and others with low patient gene sequences available in GenBank. The details of the gene dataset are displayed in Fig 2.

**Fig 3:**
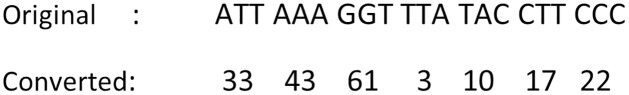
Indexing from nucleotide to the codon.

There are a few partial genes in this collected dataset. Therefore, we filtered them and only kept those that had the “complete” genome status including the reference gene sequence with a length of 197209. Finally, the dataset was reduced using a minimum gene length of 190083 and a maximum gene length of 206372. As a result, overall patient numbers drop from 757 to 512. The size of the filtered dataset resulted in the division of the mutation rates computations into three groups: Canada, Germany, and the rest of the world. Moreover, the dataset is organized in a way that makes it possible to calculate the “nucleotide mutation” and “codon mutation” separately. The nucleotide mutation rate is determined using the first filtered dataset. After that, we changed the four unprocessed nucleotides (A = adenine, T = thymine, C = cytosine, and G = guanine) into a codon set, which is a three-nucleotide unit of genetic code found in DNA or RNA. The information in Table 1 has been used in this context to transform the gene sequence by its sequence number. For example, "TTT" will be translated to 1, "GCT" will be 53, and so on. Fig 3 illustrates the conversion process. This conversion process is essential to understanding the monkeypox codon sequence mutation. Additionally, it helps to reduce computing complexity.

**Table 1:** Nucleotide conversion using the codon indexing sequence.

	T	C	A	G	
T	1. “TTT”	5. “TCT”	9. “TAT”	13. “TGT”	T
	2. “TTC”	6. “TCC”	10. “TAC”	14. “TGC”	C
	3. “TTA”	7. “TCA”	11. “TAA”	15. “TGA”	A
	4. “TTG”	8. “TCG”	12. “TAG”	16. “TGG”	G
C	17. “CTT”	21. “CCT”	25. “CAT”	29. “CGT”	T
	18. “CTC”	22. “CCC”	26. “CAC”	30. “CGC”	C
	19. “CTA”	23. “CCA”	27. “CAA”	31. “CGA”	A
	20. “CTG”	24. “CCG”	28. “CAG”	32. “CGG”	G
A	33. “ATT”	37. “ACT”	41. “AAT”	45. “AGT”	T
	34. “ATC”	38. “ACC”	42. “AAC”	46. “AGC”	C
	35. “ATA”	39. “ACA”	43. “AAA”	47. “AGA”	A
	36. “ATG”	40. “ACG”	44. “AAG”	48. “AGG”	G
G	49. “GTT”	53. “GCT”	57. “GAT”	61. “GGT”	T
	50. “GTC”	54. “GCC”	58. “GAC”	62. “GGC”	C
	51. “GTA”	55. “GCA”	59. “GAA”	63. “GGA”	A
	52. “GTG”	56. “GCG”	60. “GAG”	64. “GGG”	G

## Gene mutation

**Fig 4:**
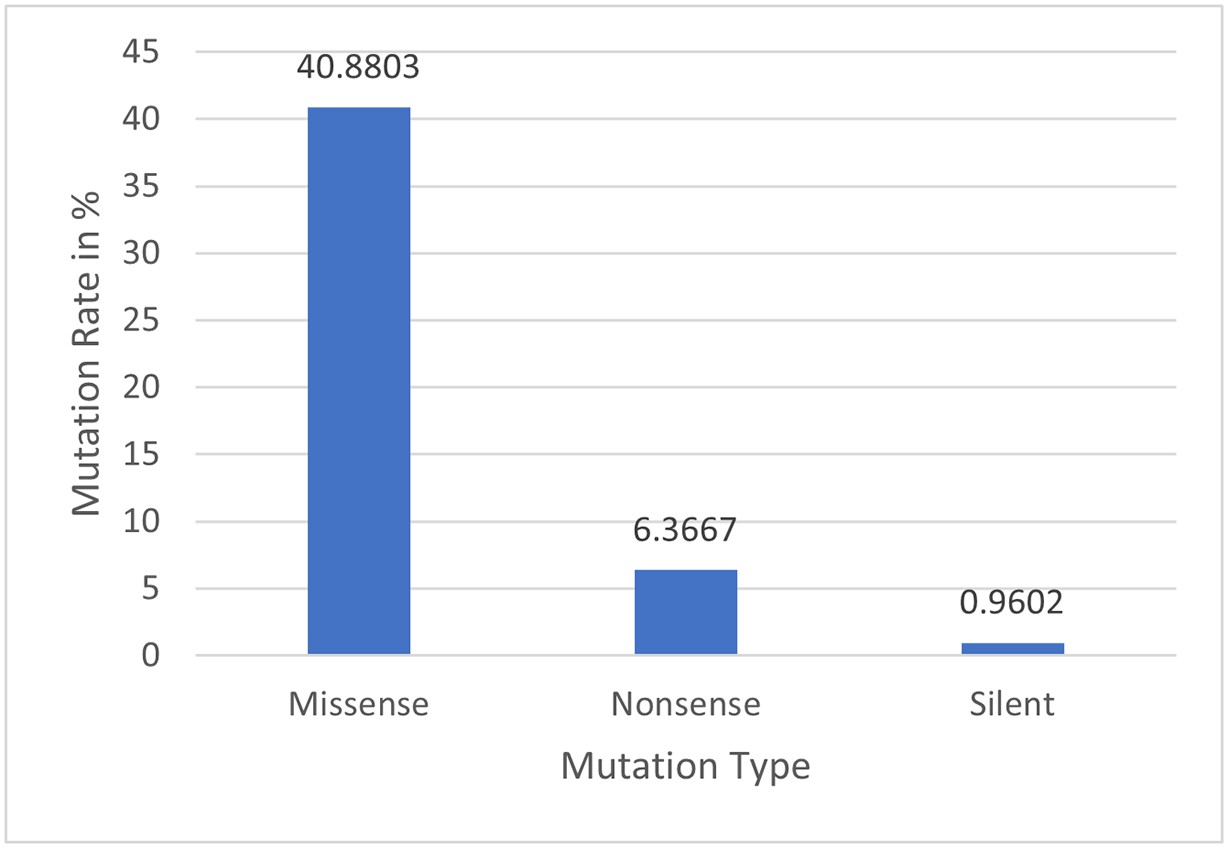
Substitution mutation rate.

Numerous factors can cause a gene to mutate. When RNA attempts to duplicate genetic information from DNA, errors may occur that lead to mutation. Additionally, the mutation is brought on by errors in DNA recombination, replication, and chemical damage to DNA or RNA. Basically, there are three different kinds of mutations: “base substitutions”, “deletions”, and “insertions”. We can determine the three types of substitution mutation present in this dataset: silent, missense, and nonsense. Silent mutations are codon changes where the resultant amino acid is left unchanged. A missense mutation is said to have occurred if the ensuing amino acid has changed. Moreover, it is referred to as a nonsense mutation when a codon changes, resulting in the gene translation being stopped, leading to an inoperable protein. The three different substitution mutation types found in the dataset are shown in Fig 4, with missense mutation rates of 40.8803%, nonsense mutation rates of 6.3667%, and silent mutation rates of 0.9602%.

### Mutation in nucleotides

**Fig 5:**
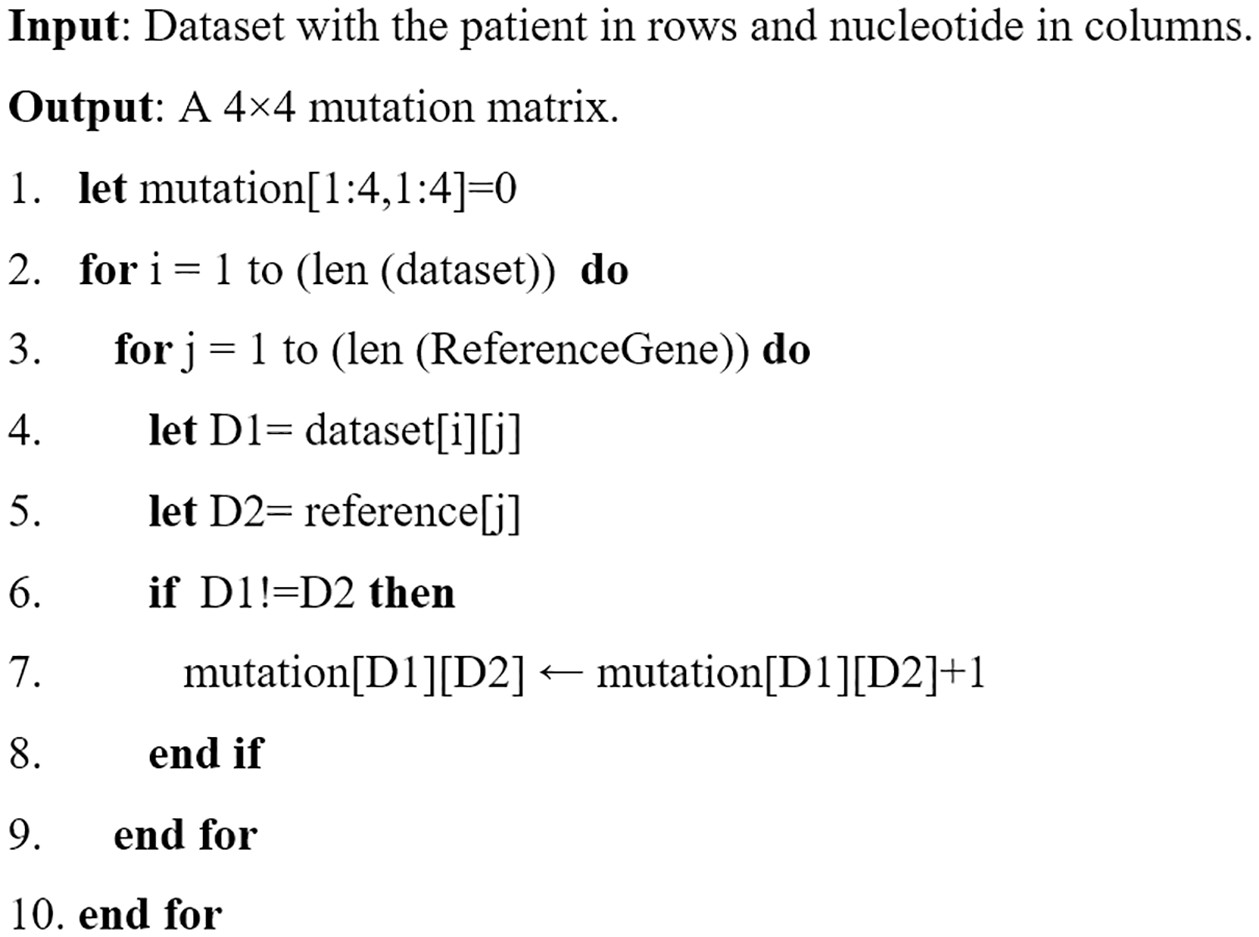
Algorithm for calculating nucleotide mutation rate.

When the type of mutation is missense, the nucleotide changes affect protein synthesis and could change the virus’s behavior. Moreover, finding the cure’s gene sequence is quite tricky. The missense nucleotide mutation rate has been established using the algorithm shown in Fig 5. After applying this algorithm, the values were converted into percentages using Eq (1).


$$MutationRate=\;\left(\frac{mutation}{\text{lg}*gs}\right)*100$$ (1)

**Fig 6:**
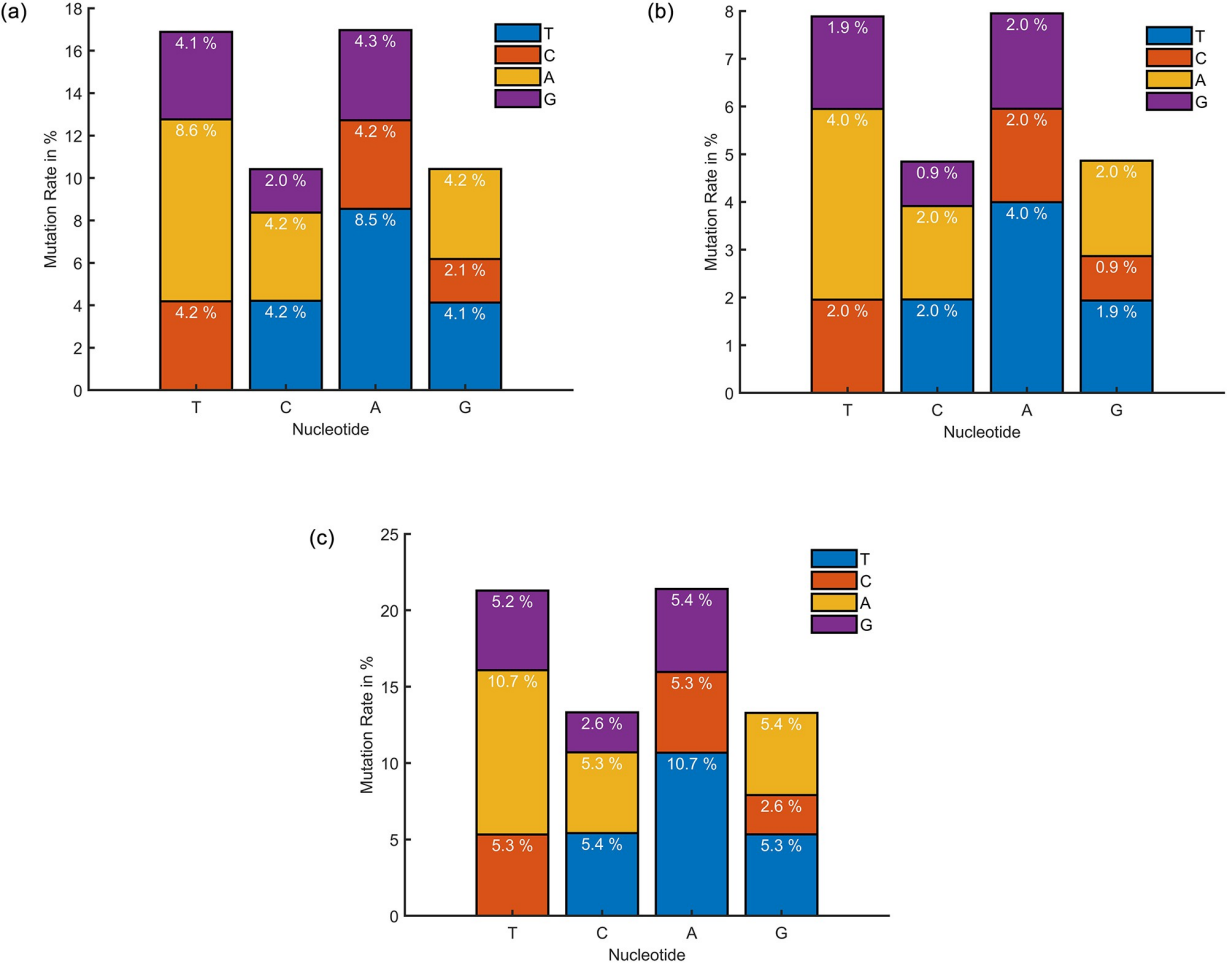
Mutation rate of nucleotide for (a) Canada, (b) Germany, and (c) the Rest of the Countries.

The final output array in this case is called "MutationRate," the output array "mutation" is of size 44 and contains the raw values received after performing the method, and the term, lg is the dataset length, which in this dataset is 512 for the total dataset, 105 for Canada, and 186 for Germany. The term gs is the length of the reference gene sequence, which in this dataset is 197209 in length. We have computed the nucleotide mutation rate for the prepared dataset in this step. The mutation rate for Canada is shown in Fig 6(a). Germany in (b) and all in (c) explain that a considerable percentage of thymine (T) and Adenine (A) is converting into other nucleotides compared with Cytosine (C) and Guanine (G). But the amount of nucleotides mutate to other types, and mutate back to their own class, which may explain why the virus is stable till now and does not have a large variety in its behavior. In comparison to Canada and Germany, the mutation rate of the overall dataset is high. Moreover, the dataset from the rest of the countries shows some variations in T, C, A, and G. Based on the availability of data from other countries, these values change.

### Codon mutation

**Fig 7:**
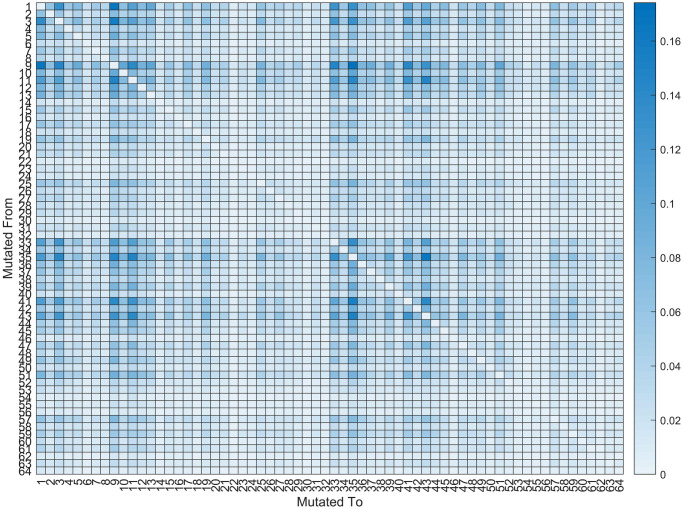
Processed mutation rate of codon for the full dataset. X and Y axis ticks are numbered following the sequence shown in Table 1.

The codon mutation rate was calculated using the second processed and converted dataset created previously, as shown in Fig 7. Modifications to the codon set result from nucleotide changes and ultimately have an immediate impact on the protein. To determine the codon mutation rate, we applied the same algorithm displayed in Fig 5. The receiving array has seen a small change, going from a nucleotide array size of 4 × 4 to a codon mutation array of 64×64. Following the codon mutations’ discovery, the percentage rates were obtained using Eq (2).


$$CodonMutation=\;\left(\frac{mutation}{\text{lg}*gs}\right)*100$$ (2)

Here, “CodonMutation” is the final output array, “mutation” is the output array with a size of 64×64 that contains original values after the algorithm is applied, lg is the dataset length, which in this converted dataset is 521, and gs is the length of the reference gene, which is 197209 in this dataset. Fig 7 shows the codon mutation rate for the entire dataset. It is evident from the obtained value that codons do not frequently mutate in the same way as nucleotides do. The diagonal values are 0 since the maximum codon mutation rate is 0.174% and the point codons are not changing compared to the reference gene.

## Mutation rate prediction for nucleotide

**Fig 8:**
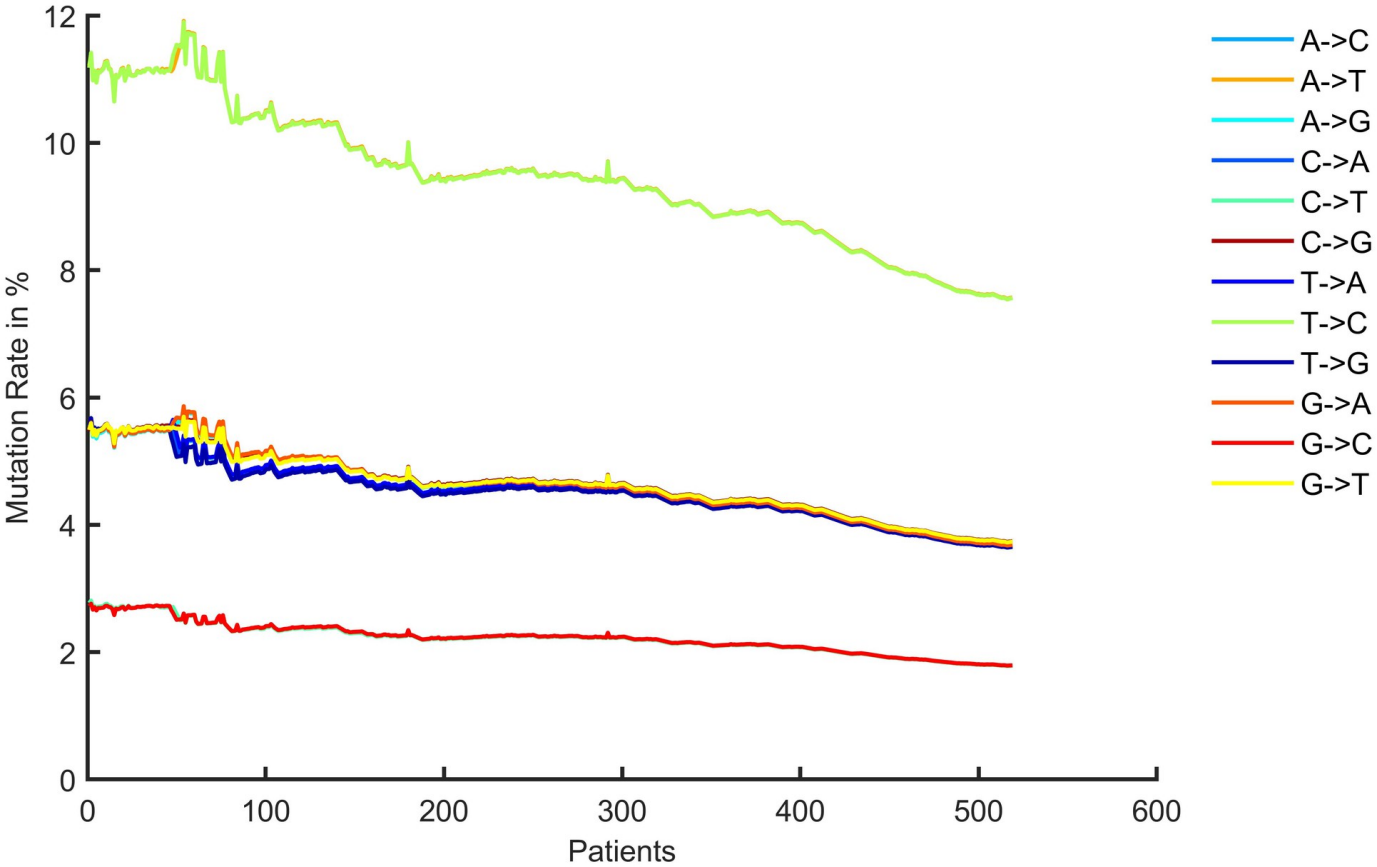
Time series dataset based on patients.

The processed nucleotide dataset contains data that includes the period from 12th December 2001 to 16th May 2022 in a discontinuous manner. Since, dates are arranged in ascending order at the data pre-processing stage, it is simple to process this as a time series dataset. This dataset contains one or more patients for one specific date. By collecting all the patients, we created the time series sequential dataset for patients which is shown in Fig 8.

**Fig 9:**
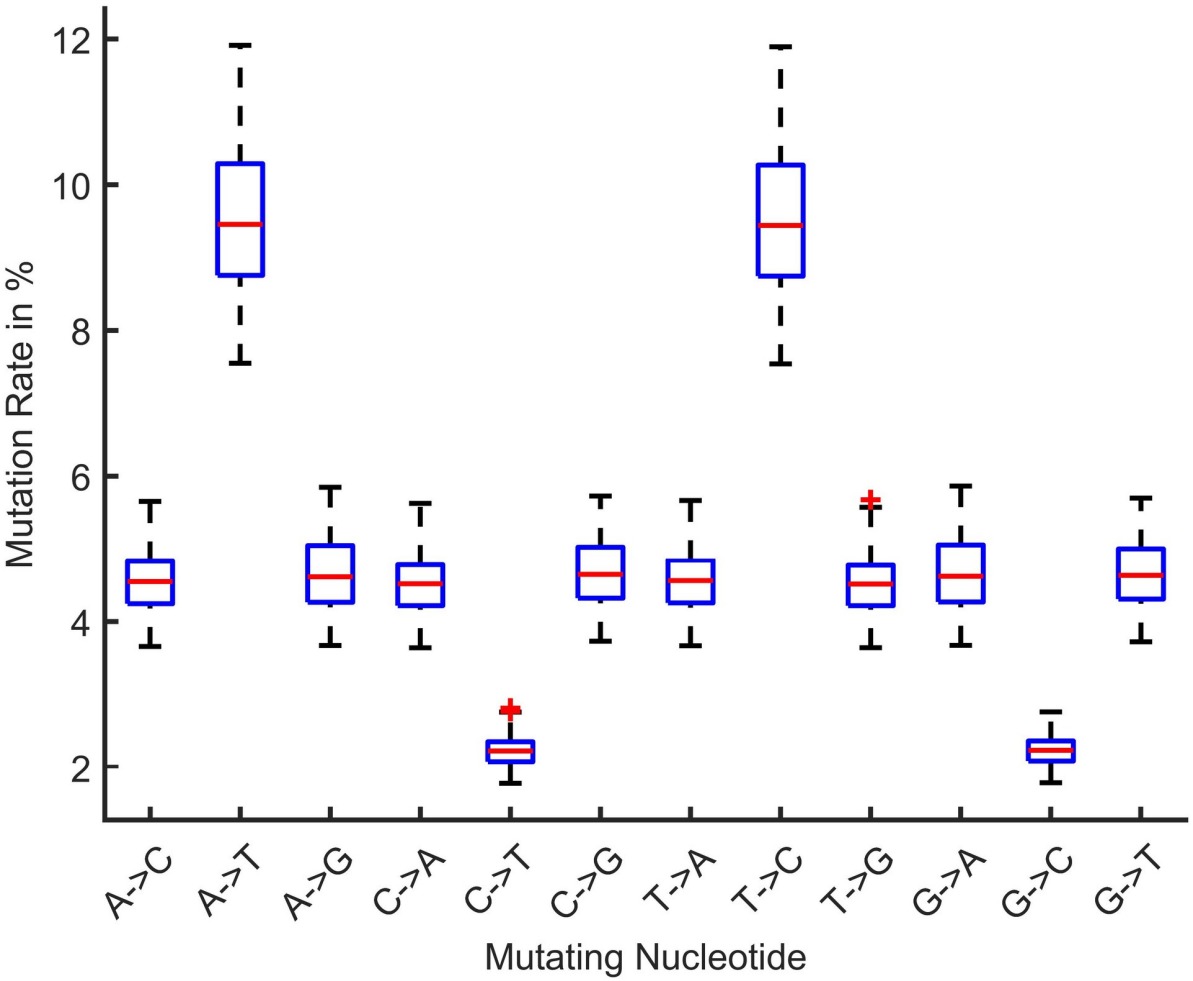
Mutation rate limits for the available timeframe.

In order to obtain a daily time-series dataset, we estimated the mean mutation rate for various patients on the same date to prevent overlapping concerns. The dataset becomes smaller, as a result, the dates are arranged in a non-sequential ascending order Fig 9 displays the mutation rates for the entire timeframe. Due to the minimal availability of data, it is difficult to train a model on such a tiny amount of data.

A deep learning-based LSTM network has been used in this experiment. Instead of taking one patient per step, we utilized it as a set of 12 patient per step and the format have been presented in Table 2.

**Table 2:** Training data preparation.

Data (12×12)	Label (1×12)
Processed dataset indexed set {1, 2, 3 …..11, 12}	Corresponding target index 13
Processed dataset indexed set {2, 3, 4 …..12, 13}	Corresponding target index 14
Processed dataset indexed set {3, 4, 5 …..13, 14}	Corresponding target index 15
Processed dataset indexed set {n-12, n-11, n-10 …… n-2,n-1}	Corresponding target index n

**Fig 10:**
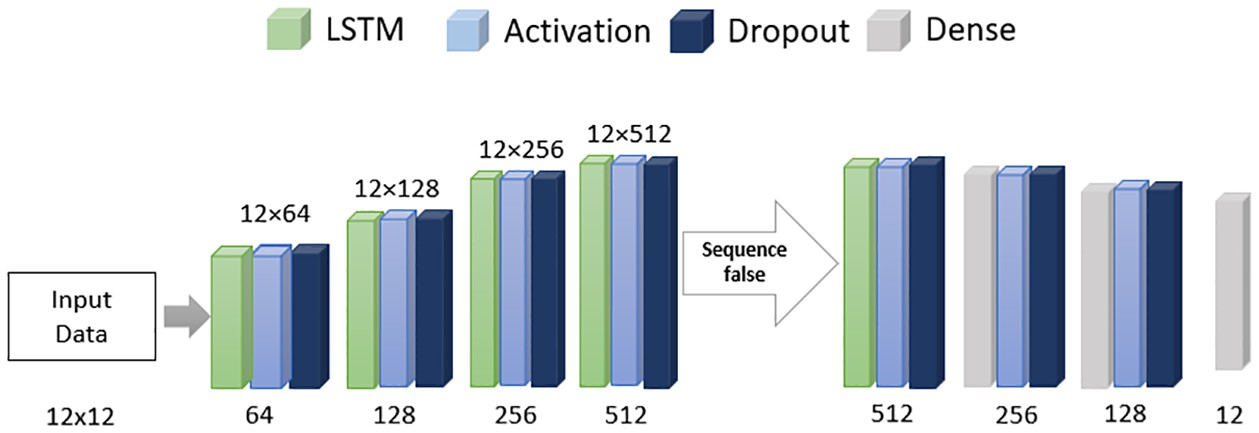
The LSTM model architecture used for the train time series dataset.

As for training and testing data, the entire set of data has been split 90–10%, respectively as the dataset is low. As a result, we got 467 rows for training and 52 for testing. To train the dataset, an LSTM model has been built using the Python deep learning API Keras and its structure is shown in Fig 10. The model contains a stack of LSTM layers with 64, 128, 256 and 512 units, linear activation functions and 10% dropout. After the flatten equivalent layer where LSTM does not return any sequence, the unit reduces to 256, 128 and finally to 12 to maintain the data shape. With adam optimizer and 100 epochs have been used with a tensor board to track the losses. In testing and training, this model’s RMSE values are 0.09 and 0.08, respectively. For GRU, we used a simple model architecture consisting of (12,12) input shape, followed by bidirectional GRU layer with 32-unit, tanh activation, sigmoid recurrent activation as encoder layer. Then used RepeatVector layer with 12 unit, again followed by a bidirectional GRU with 32 unit used as a decoder layer. And finally, uses TimeDistributed Dense layer with linear activation.

**Fig 11:**
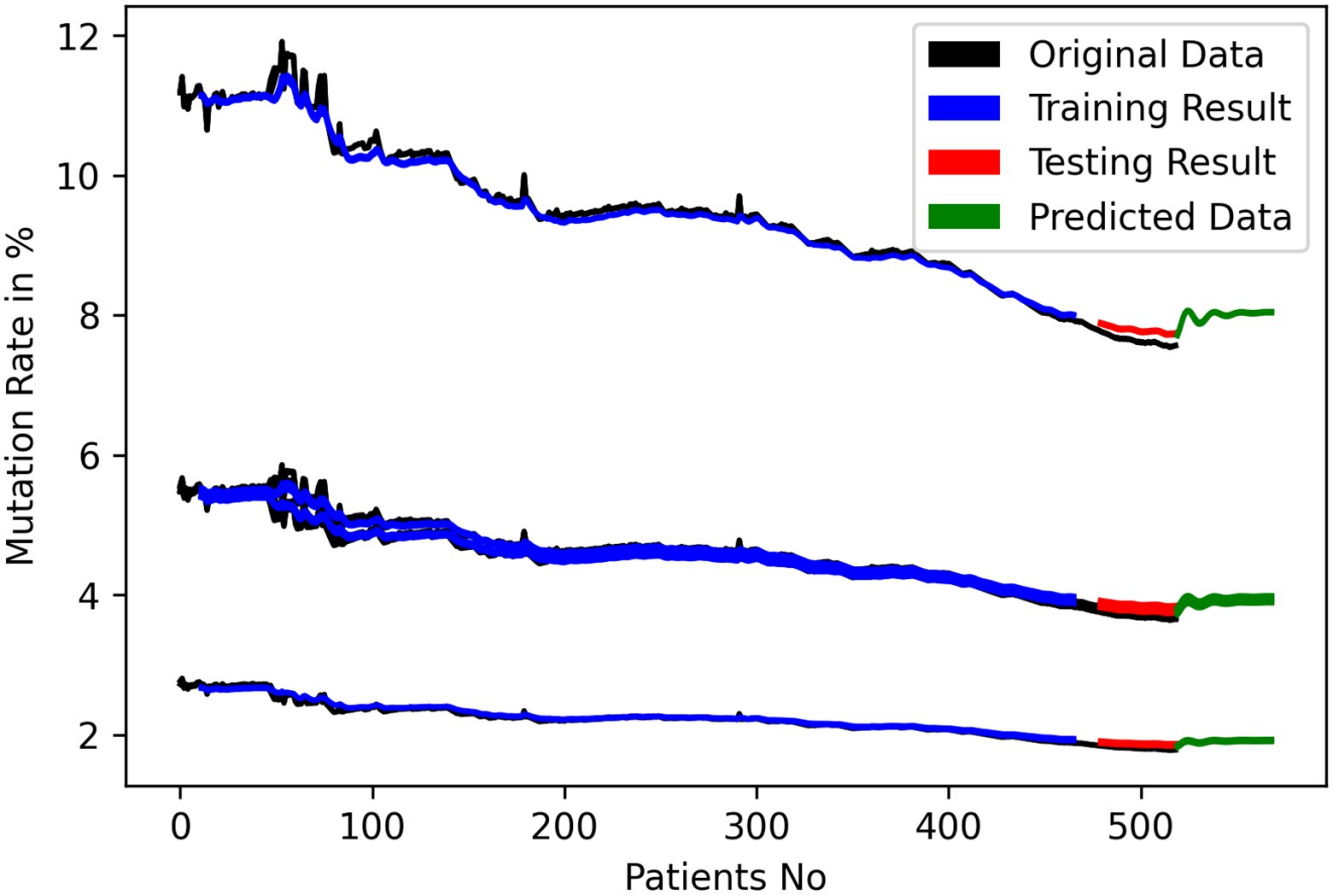
Training, validation and nucleotide rate prediction with LSTM.

**Fig 12:**
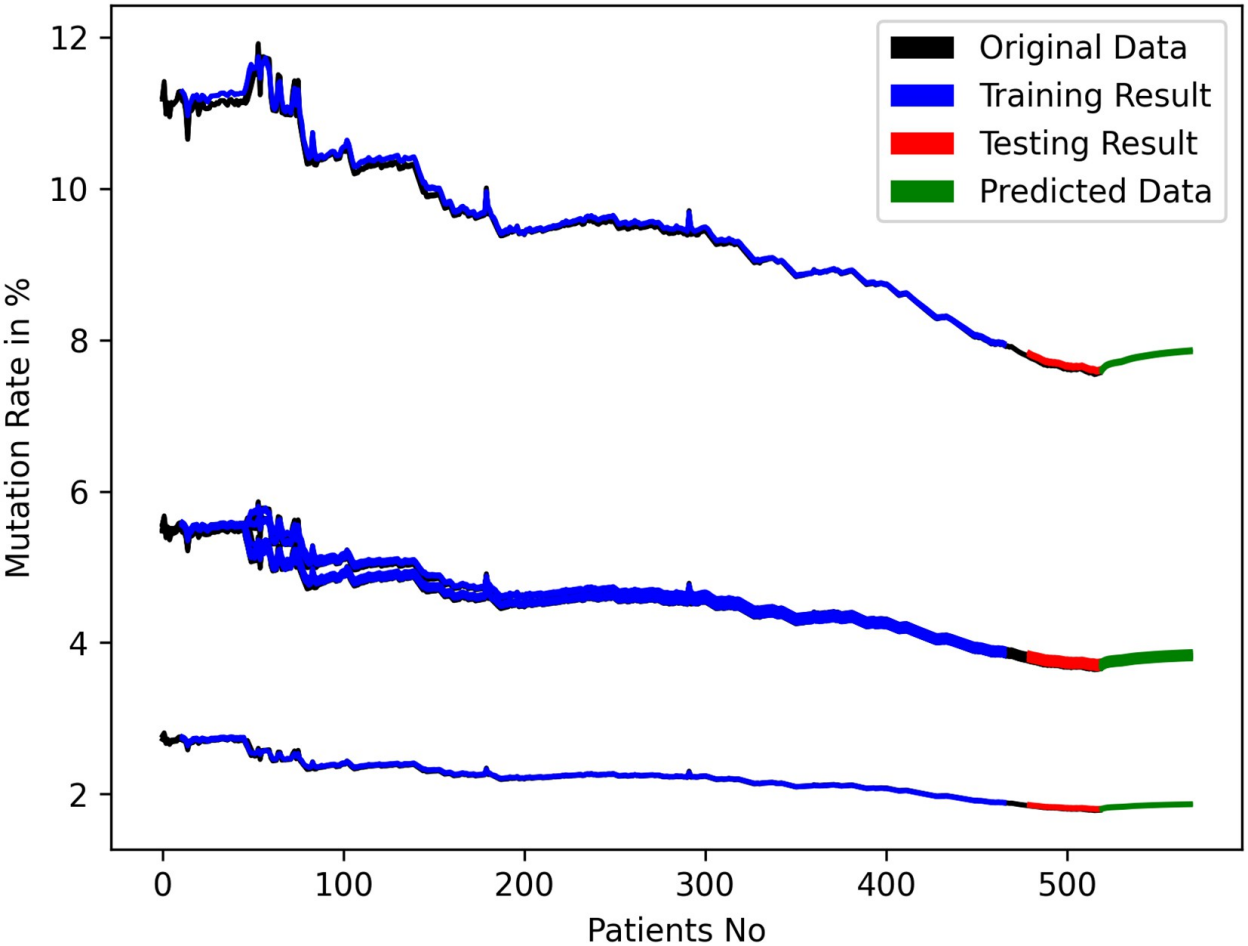
Training, validation and nucleotide rate prediction with GRU.

After the training and testing phase, it was discovered that both models were performing at the expected level. Therefore, we predicted each future patient’s gene mutation rate using the mutation rates of the previous 12 patients. We then took that patient and calculated the mutation rates of the previous 12 patients using 11 old patients and 1 new patient. Using this method, we were able to predict the future mutation rates for 50 patients using the LSTM, as shown in Fig 11. For GRU, we used n^th^ patient data to predict (n+1)^th^ future patient and the predicted mutation rates are shown in Fig 12.

**Fig 13:**
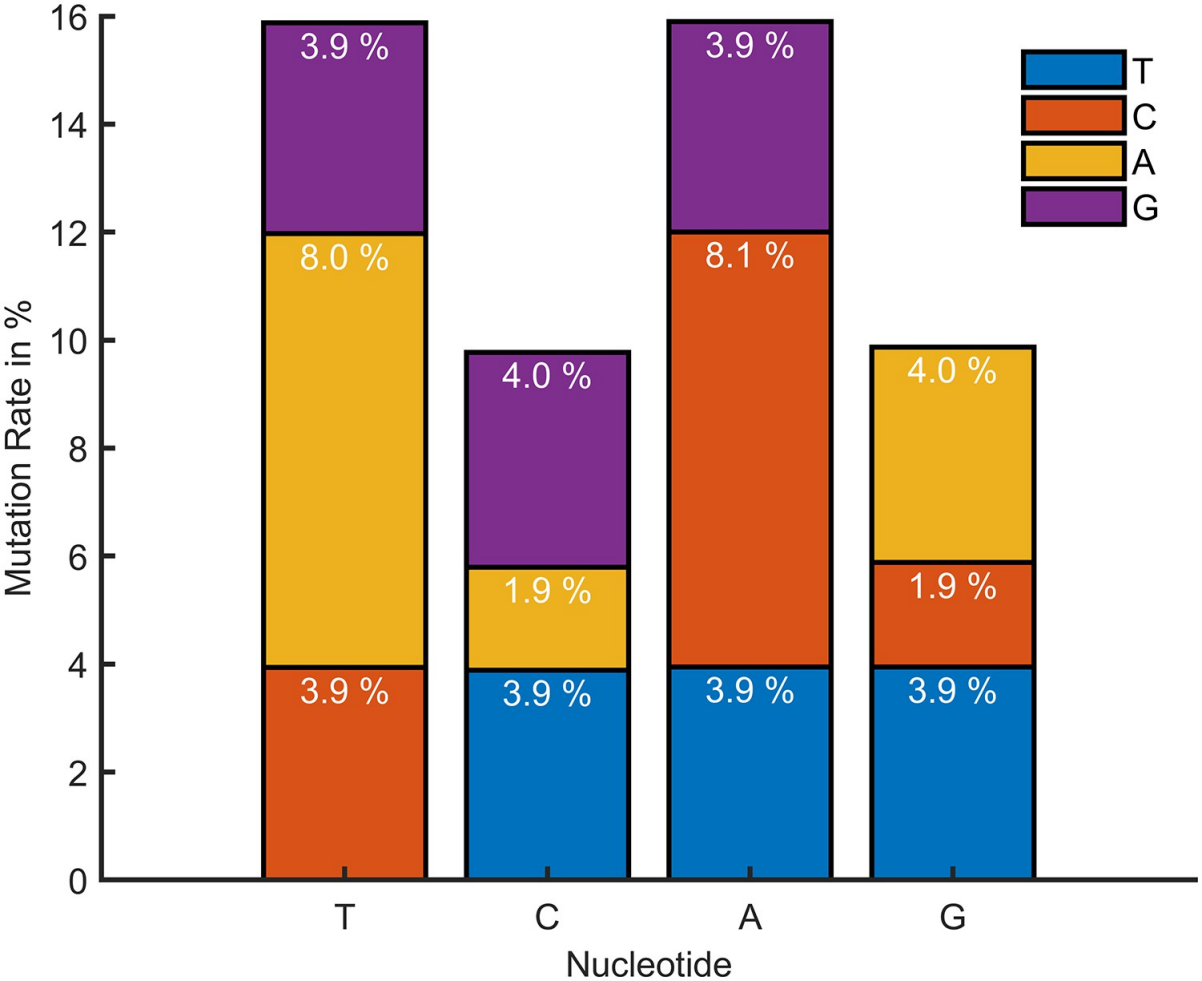
Mutation rate prediction for 50th future patient for LSTM.

**Fig 14:**
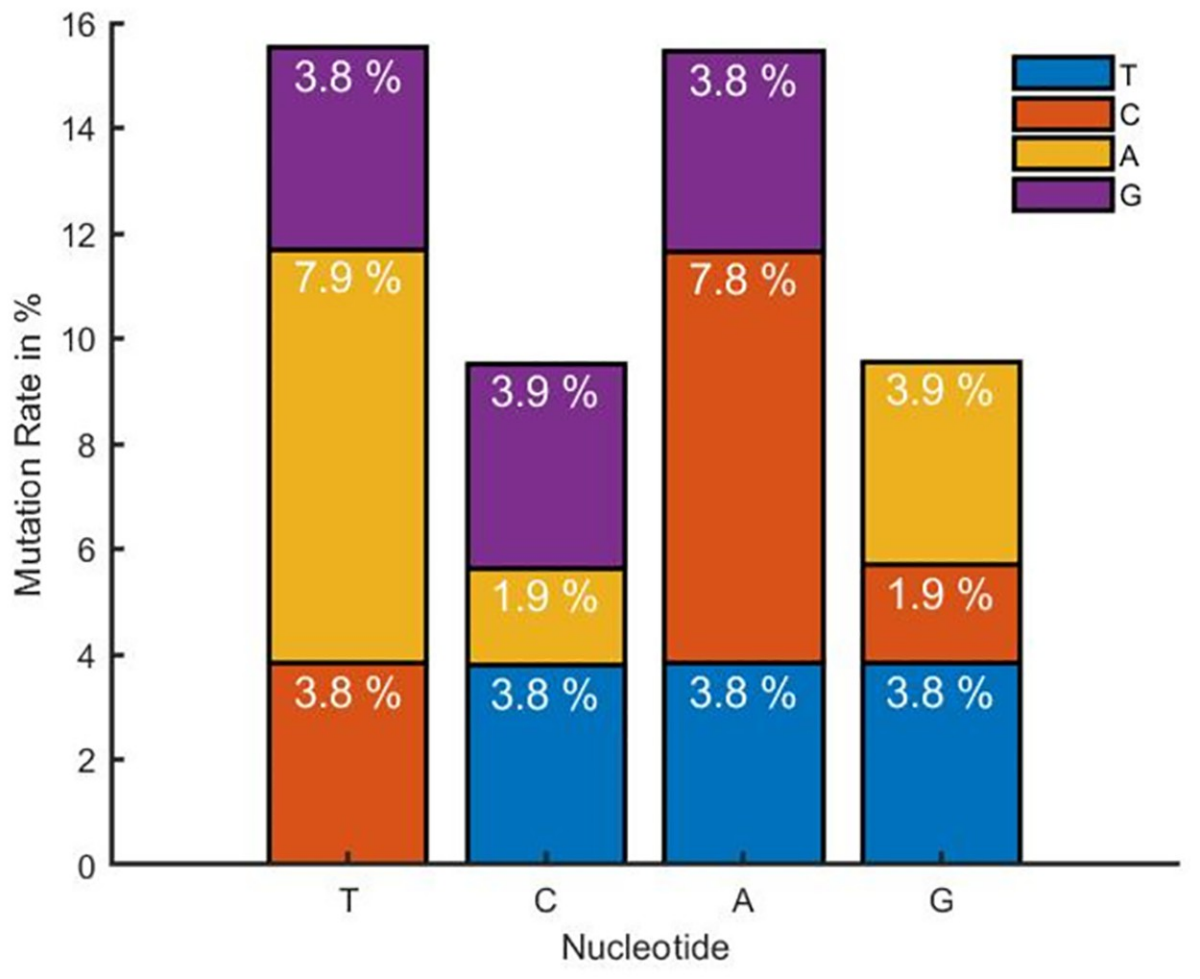
Mutation rate prediction for 50th future patient for GRU.

Fig 13 shows the mutation rate of nucleotide for the 50^th^ patient in the near future time for LSTM and Fig 14 shows for GRU. The mutation rate appears to have marginally decreased. The balance between forward and backward mutation has been seen. The amount of nucleotide mutated from T→C and C→T; T→G and G→T are almost equal, T→A and A→T differ by 0.1%, the big difference of 6.2% is seen for C→A and A→C, C→G and G→C are also differ by 2.1%, 0.1% difference has been seen between A→G and G→A. If additional continuous data can be collected from different geo-locations and periods, this approach can be used to calculate the mutation rate for a certain date in the future.

### Comparison with similar literature work

A similar kind of work has been found for COVID-19 gene mutation. Still, this work is significant for monkeypox gene analysis as no other literature has been found yet. We are working on a real-time dynamic system to process and train the latest data automatically because the patient’s data gradually increases in the NCBI dataset. Updating the whole work based on those new data is time-consuming. The LSTM modal that we used is custom, simple and lightweight for training and we have used google colab with GPU. This modal can predict the most probable mutation rate scenario that might be seen in future patients. We also trained GRU as a validation comparison with LSTM so that predictions can be validated. Compared with nucleotide mutations, the GRU predicted nearly same result as LSTM with slight 0.01% difference. Another recent study showed that the substitutional mutation rate is 38.63 worldwide for 2022, where our result is 40.88 which is slightly higher than their result, due to having less data in our study.

Many have previously worked with COVID-19 mutation rate analysis, and based on that result, it can be mentioned that, this model is working as expected and no overfitting is noticed. Although some research on monkeypox was done where distinct forecasting techniques were utilized, suggesting that the Prophet model is the most accurate forecasting model [9]. Moreover, other existing works showed the prediction of monkeypox cases based on the LSTM, MLP, and ARIMA models [16, 28]. However, the central fact is that those types of work are not with gene mutations. As a result, our work is unique in the perspective of time series analysis of the monkeypox mutations.

## Conclusion

The monkeypox virus’s severity and wide spreadness in this year attracted attention. Already several works have been done on this analysis and forecast for the future events of this virus except for gene mutation. Genetic data has been used by the latest analysis techniques to understand the importance of an object’s behavior. With the upgrade of computing power and algorithms, we can now see the future based on numbers. LSTM has been widely used and is one of the most popular models to predict time series data. This paper uses an LSTM model to train and predict monkeypox mutation on substitutional patients’ processed dataset. Using this, the 50^th^ future mutation rate has been predicted, and a lower rate is noticed. Also, the codon rate is shown to understand the flow of change at the protein level. As the dataset is comparatively lower than the covid case, it was impossible to go further. But if the number of patients increases, we expect more gene data collection in the NCBI database.
